# Introduction of Exogenous HSV-TK Suicide Gene Increases Safety of Keratinocyte-Derived Induced Pluripotent Stem Cells by Providing Genetic “Emergency Exit” Switch

**DOI:** 10.3390/ijms19010197

**Published:** 2018-01-09

**Authors:** Maciej Sułkowski, Paweł Konieczny, Paula Chlebanowska, Marcin Majka

**Affiliations:** Department of Transplantation, Faculty of Clinical Immunology and Transplantation, Institute of Pediatrics, Collegium Medicum Jagiellonian University, Wielicka 265, 30-663 Kraków, Poland; maciek.sulkowski@uj.edu.pl (M.S.); pawel.konieczny@outlook.com (P.K.); paulalota0@gmail.com (P.C.)

**Keywords:** induced Pluripotent Stem (iPS), suicide gene therapy, genetic safeguarding

## Abstract

Since their invention in 2006, induced Pluripotent Stem (iPS) cells remain a great promise for regenerative medicine circumventing the ethical issues linked to Embryonic Stem (ES) cell research. iPS cells can be generated in a patient-specific manner as an unlimited source of various cell types for in vitro drug screening, developmental biology studies and regenerative use. Having the capacity of differentiating into the cells of all three primary germ layers, iPS cells have high potential to form teratoma tumors. This remains their main disadvantage and hazard which, until resolved, prevents utilization of iPS cells in clinic. Here, we present an approach for increasing iPS cells safety by introducing genetic modification—exogenous suicide gene *Herpes Simplex Virus Thymidine Kinase* (*HSV-TK*). Its expression results in specific vulnerability of genetically modified cells to prodrug—ganciclovir (GCV). We show that HSV-TK expressing cells can be eradicated both in vitro and in vivo with high specificity and efficiency with low doses of GCV. Described strategy increases iPS cells safety for future clinical applications by generating “emergency exit” switch allowing eradication of transplanted cells in case of their malfunction.

## 1. Introduction

Since their invention, induced Pluripotent Stem cells (iPS) [1,2], remain a great promise for regenerative medicine. Their greatest advantage is a possession of all traits of Embryonic Stem cells (ES) [3] while being free of their main limitation—ethical issues concerning disruption of human embryos during isolation. iPS cells are generated by cellular reprogramming of somatic cells into pluripotency [4] and can be obtained from virtually any cell type. This also implies that they can be created in a patient-specific manner and are able to deliver autologous cells of interest for transplantation. iPS cells enable generation of patient-derived in vitro models, which can serve for personalized drug screening [5,6]. Patient-specific iPS cells were generated for Parkinson’s disease, Huntington’s disease, Gaucher’s disease, Becker’s and Duchenne’s muscular dystrophy, Down’s syndrome, amyotrophic lateral sclerosis, LEOPARD syndrome, diabetes type I and other disorders [7,8,9,10]. It was shown that iPS-derived somatic cells recapitulate symptoms/phenotypes of diseases in vitro [11,12,13] thus offering a personalized model for in vitro drug screening. Moreover, they were also able to improve symptoms in numerous animal models of diseases [7,8,9]. 

Originally, iPS cells were generated by viral transduction [1,2]. Such strategy is not optimal as it generates risk of uncontrolled insertional mutagenesis. Thus, several safer, non-integrating methods have been proposed since 2006. These include use of recombinant proteins [14,15], transposons [16,17] mRNA [18], DNA plasmids [19], non-integrating viruses [20] or chemical stimulation [21,22] (reviewed in [23]). These reprogramming methods increase safety of iPS cells.

Despite of all advantages of iPS cells, they still cannot be used in clinic until their major drawback—tumorigenesis risk—is eliminated. As pluripotency is a double-edged sword, iPS cells are able to form teratomas—tumors built of all three germ layers [24,25,26]. A possible approach to overcome this issue is to safeguard iPS cells by equipping them with suicide gene which selectively sensitizes genetically modified cells to normally non-toxic compounds. An example of such strategy is suicide gene therapy with Herpes Simplex Virus Thymidine Kinase (HSV-TK) and its substrate, antiviral medication, ganciclovir (GCV, 9-[(1,3-dihydroxy-2-propoxy)methyl]guanine). This approach was proved to be highly effective anti-cancer therapy in vitro [27,28,29] and in vivo [30,31] as well as in a clinical trial [32].

Thymidine kinase (EC 2.7.1.21) is the key phosphotransferase in the pyrimidine salvage pathway catalyzing specific transfer of γ-phosphate from ATP to thymidine producing dTMP [33]. It is present in two forms in most mammalian cells. Viral HSV-TK, introduced into mammalian cells during viral infection, unlike specific cellular thymidine kinase, catalyzes reactions of various substrates including pyrimidine analogs (such as thymidine and deoxycytidine), and purine analogs (acyclovir, ganciclovir, buciclovir and penciclovir). GCV is phosphorylated to GCV-monophosphate by HSV-TK and further by cellular kinases to GCV-triphosphate, which is a competitive inhibitor of deoxyguanosine triphosphate (dGTP) and incorporates into DNA inhibiting DNA replication [34]. Additionally, toxic GCV-triphosphate can be efficiently transferred to adjacent cells resulting in elimination of unmodified cells of the same type in, so called, bystander effect [35]. Transport of active GCV metabolite to adjacent cells is mediated by gap junctions—intercellular channels built from connexins—which allows direct diffusion of ions and small molecules (of mass up to 2 kDa) between neighboring cells [36,37]. This transfer induces similar susceptibility of adjacent cells to GCV and can be so efficient that as few as 10% of malignant cells expressing *HSV-TK* gene in population is sufficient to eliminate all tumor cells [35].

Here, we present a strategy to increase iPS cells safety by incorporating “safety switch” or “emergency exit” which allows efficient elimination of all iPS cells genetically modified to express HSV-TK by simple addition of non-toxic prodrug GCV when needed. We generated iPS cells by reprogramming plucked-hair keratinocytes using safe method—transduction with non-integrating Sendai virus vector. Generated keratinocytes-derived-iPS (kiPS) cells were characterized and subsequently genetically modified with lentiviral vectors harboring *HSV-TK*. For proof-of-concept studies, lentiviral transduction was used, however in future studies this approach will be replaced with safer method of transgene delivery. iPS cells expressing HSV-TK were efficiently eliminated both in vitro and in vivo upon administration of CGV. This proves efficiency of suicide gene therapy of iPS cells and feasibility to eradicate all genetically modified transplanted cells upon their “rogue” behavior.

## 2. Results

### 2.1. Reprogramming of Keratinocytes from Plucked Hair

**Figure 1:**
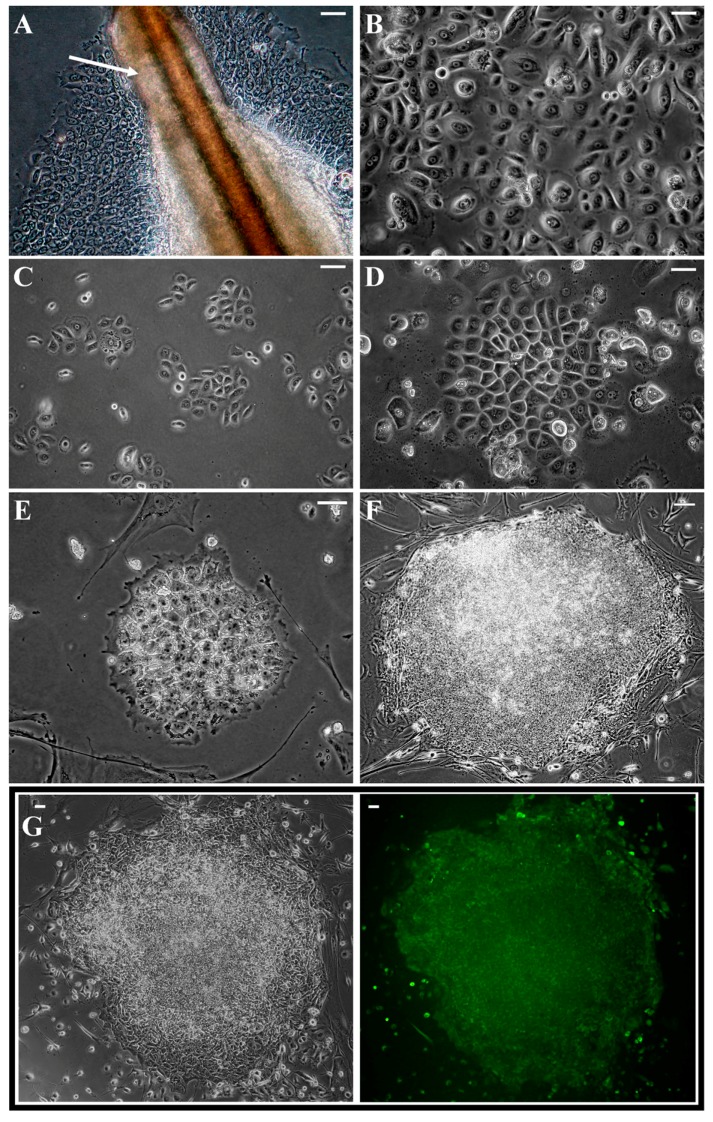
Isolation and reprogramming of plucked-hair keratinocytes: (**A**) keratinocytes migrating from plucked-hair outer root sheath (ORS), ORS is marked with white arrow; (**B**) morphology of isolated keratinocytes after their subculture and expansion; (**C**) isolated keratinocytes one day before infection; (**D**) morphology of transduced keratinocytes changed to epithelial within four days after infection; (**E**) first iPS-like colonies appeared around Day 14 post infection; (**F**) typical morphology of transferred colonies on Days 28–35; (**G**) colonies expressed alkaline phosphatase (green fluorescence); (**A**–**E**) magnification 200×, white bars represent 50 µm; and (**F**,**G**) magnification 10×, white bars represent 100 µm.

Keratinocytes from plucked hair seem to be very promising cell type for reprogramming. Acquisition of hairs is simple and non-invasive. Between ten and twenty hairs were obtained from healthy volunteers. Sufficient number of cells for reprogramming by direct outgrowth from hair outer root sheath (ORS), marked with arrow in Figure 1A, were obtained in 2–3 weeks. Isolated cells were expanded (Figure 1B) and cryopreserved. To achieve the highest safety of cellular reprogramming, we used non-integrating Sendai virus-based method. Within four days after infection (Figure 1C), morphology of infected keratinocytes altered and became more epithelial (Figure 1D), which is an indication of mesenchymal-to-epithelial transition occurring in early stages of reprogramming [38]. During first days after infection moderate cytotoxicity was observed. First colonies with morphology typical for pluripotent cells, i.e., tightly packed small cells with high nucleus/cytoplasm ratio, appeared around 14 days post infection (Figure 1E). These colonies quickly stopped dividing and cells dispersed suggesting that they were incompletely reprogramed pre-iPS colonies. After Day 18, fully reprogrammed iPS colonies started to appear.

Twelve colonies with most iPS-like morphology (Figure 1F), growth rate and expression of alkaline phosphatase (Figure 1G) were transferred to separate dishes between Day 28 and Day 35 post infection. Ten colonies gave start to keratinocytes-derived-iPS (kiPS) cell lines. This implies efficiency of reprograming at a level of 0.01%, which is lower than described for Sendai virus reprograming of neonatal fibroblasts. Acquired kiPS cell lines were expanded until passage 10 before subsequent analyses [20].

### 2.2. Characteristic of Acquired kiPS Cell Lines

**Figure 2:**
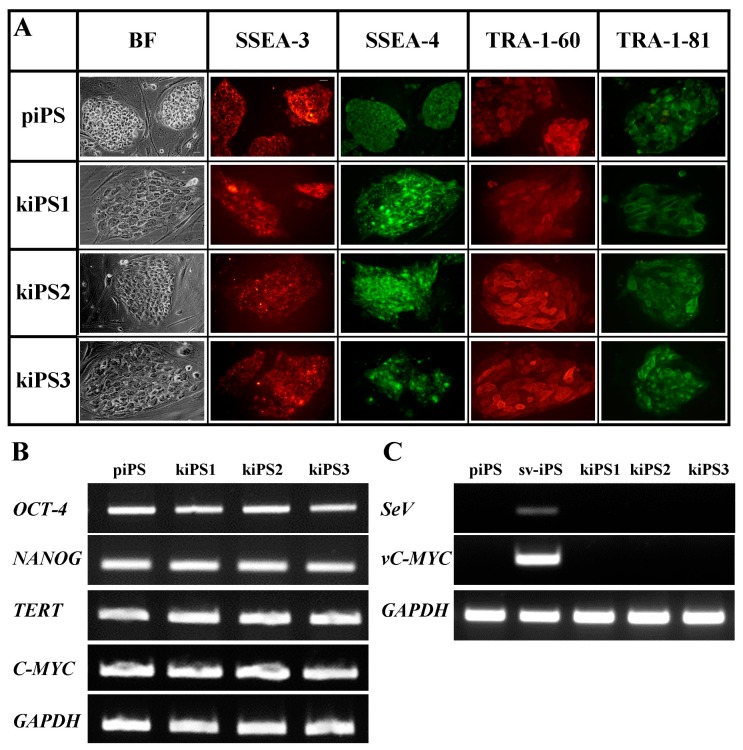
Acquired keratinocytes-derived-iPS (kiPS) cell lines express endogenous pluripotency markers and silence viral transgenes: (**A**) kiPS cell lines expressed surface antigens, i.e., SSEA-3, SSEA-4, TRA-1-60, and TRA-1-81; and (**B**) pluripotency genes, i.e., *OCT-4*, *NANOG*, *TETR*, *C-MYC*, characteristic for pluripotent stem cells. (**C**) Viral transgenes (*SeV*, *vC-MYC*) in generated kiPS cell lines were silenced at passage 15. piPS, commercially available protein-iPS cell line; sv-iPS, iPS cell line on early passage generated with Sendai Virus; *SeV*, primers specific for Sendai Virus genome; v*C-MYC*, primers specific for viral transgenic *C-MYC*; *TERT*, telomerase; *GAPDH*, housekeeping gene, glyceraldehyde 3-phosphate dehydrogenase; BF, bright field. All images in (**A**): magnification 400×, white bars represent 50 µm.

Three out of ten acquired kiPS cell lines were examined for their pluripotency markers and teratoma formation ability. All analyzed cell lines possessed high expression of embryonic pluripotency surface markers (SSEA-3, SSEA-4, TRA-1-60 and TRA-1-81) as compared to positive control—commercially available and characterized protein-iPS (piPS ) line (Figure 2A). They also possessed characteristic morphology—colonies of tightly packed small cells with large nuclei and distinct borders—and unlimited growth potential (cultured for at least 50 passages). 

Obtained cell lines also possessed endogenous expression of pluripotency markers on the level of mRNA, namely *OCT4*, *NANOG*, *C-MYC*, and telomerase (*TERT*) (Figure 2B). Viral transgenes (Sendai virus genome, *SeV* and *vc-Myc*) were silenced at passage 15 suggesting total loss of these viral transgenes at this point (Figure 2C) and fully endogenic expression of pluripotency genes.

**Figure 3:**
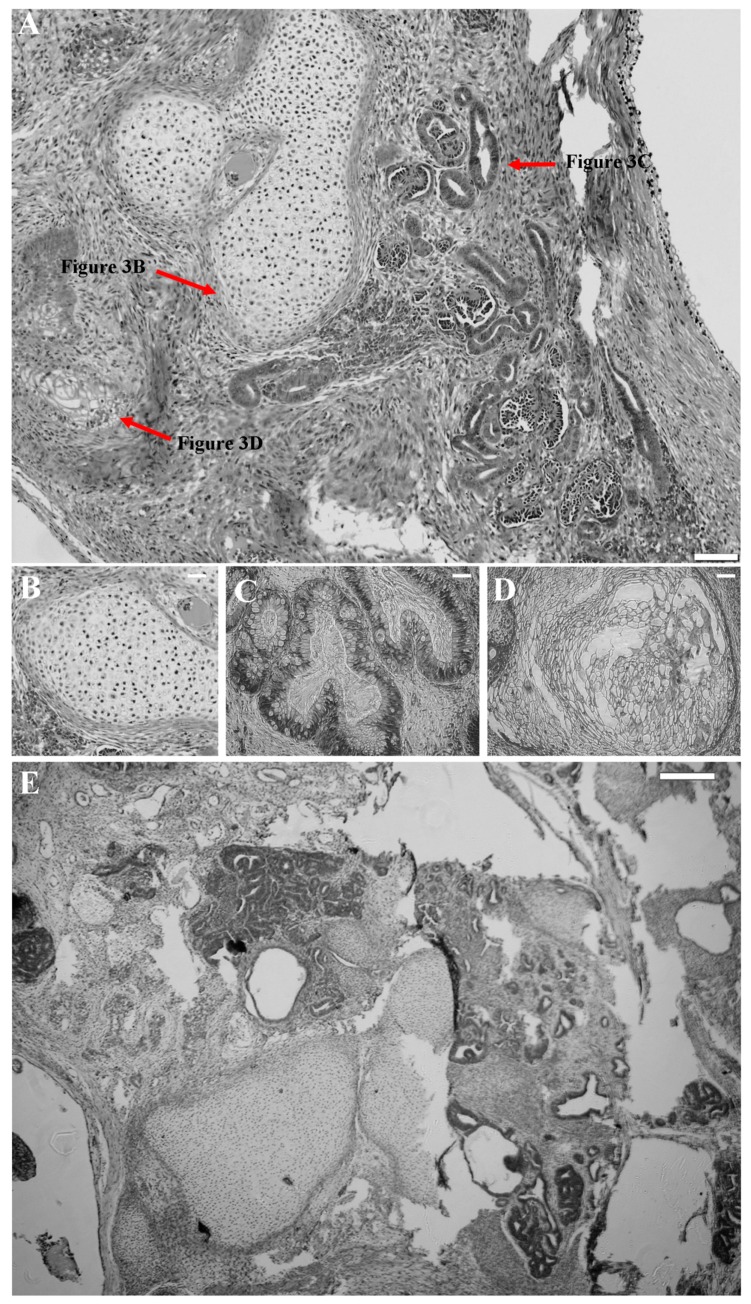
Reprogrammed kiPS cell lines generate teratomas in vivo. (**A**) Typical histopathologic images of kiPS-formed teratoma, containing three germ layer derivatives: (**B**) cartilage; (**C**) secreting epithelium; and (**D**) stratified epithelium; and (**E**) control histopathologic image of piPS-formed teratoma. (**A**–**D**) Magnification 100×, white bar represents 250 µm; and (**E**) magnification 40×, white bar represents 200 µm.

Moreover, acquired kiPS cell lines could form teratomas in immunodeficient mice (Figure 3A). Histopathological analysis of tumors revealed presence of all three germ layers. Structures characteristic for cartilage (Figure 3B), secreting epithelium (Figure 3C), and stratified epithelium (Figure 3D) could be observed within generated tumors proving pluripotency of kiPS cell lines. Characteristic image of teratoma formed by kiPS (Figure 3A) cell line was similar to one formed by control piPS cell line (Figure 3E).

These results suggest successful reprograming of keratinocytes and full pluripotency of established vector-free kiPS cell lines.

### 2.3. Genetic Modification of kiPS Cell Lines

**Figure 4:**
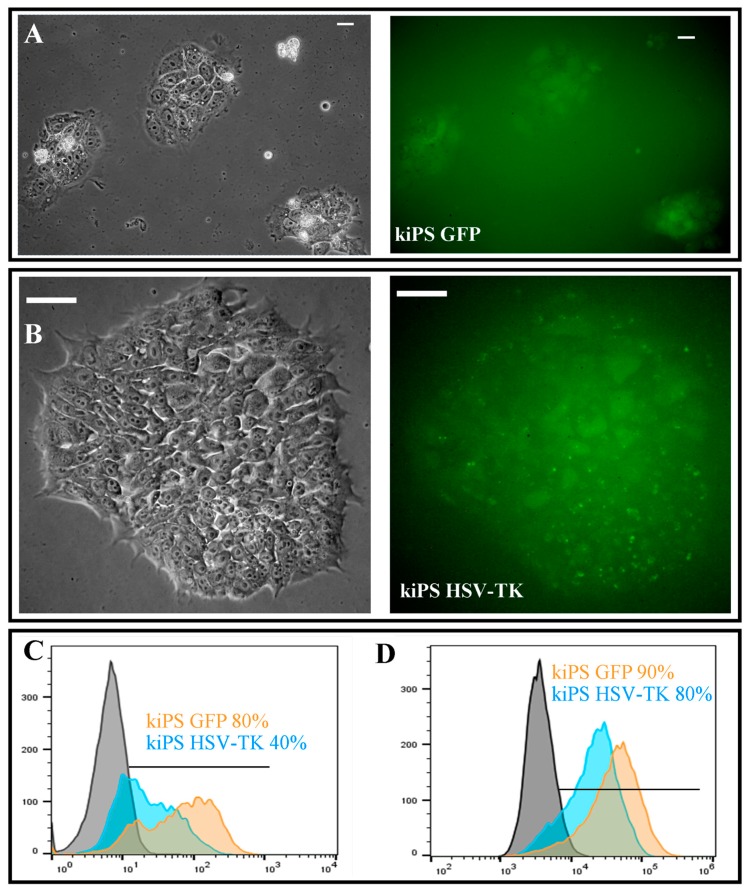
Genetic modification of kiPS cell lines. Genetically modified kiPS cell line express: GFP (**A**); or GFP and HSV-TK (**B**). (**C**) Purity of genetically modified cell lines after antibiotic selection analyzed by flow cytometry. (**D**) Purity of genetically modified cell after cell sorting. (**A**) Magnification 200×, white bars represent 50 µm; and (**B**) magnification 400×, white bars represent 50 µm.

kiPS cells were infected using lentiviral particles introducing *GFP* as a control and *GFP*-ires-*HSV-TK* construct with MOI = 3 and selected for two weeks. Established cell lines kiPS GFP (expressing GFP, Figure 4A) and kiPS HSV-TK (expressing GFP and HSV-TK, Figure 4B) were developed with moderate purity of 40% and 80%, respectively (Figure 4C). To increase purity of genetically modified population for in vivo studies, GFP-positive cells were sorted by FACS Aria and purity of at least 80% was achieved for both cell lines (Figure 4D). It is noteworthy that genetic modification did not alter the morphology of kiPS cell lines and only slightly decreased proliferation rate of kiPS HSV-TK cell line both in vitro and in vivo visible in supplementary Figure S1. We assumed that the phenomenon was caused by presence of exogenous kinase (HSV-TK) which might non-specifically phosphorylate multiple cellular targets. We observed similar phenomenon in rhabdomyosarcoma cell line [29].

Acquired data suggest efficient introduction of HSV-TK and GFP expression into established kiPS cell lines.

### 2.4. Successful Suicide Gene Therapy of kiPS Cells In Vitro an In Vivo

**Figure 5:**
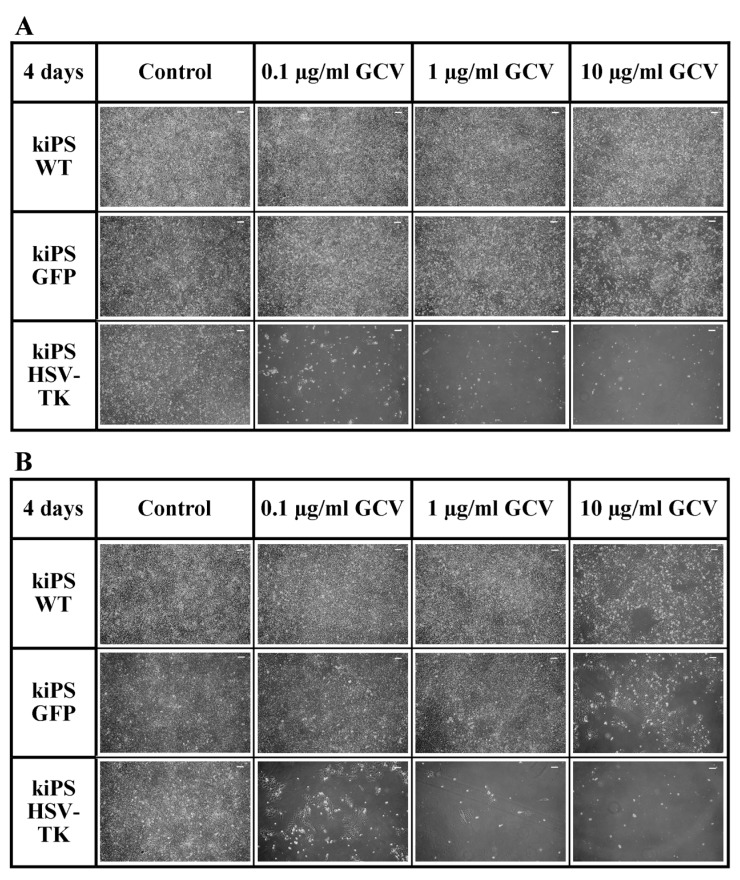
Ganciclovir efficiently eliminates HSV-TK expressing kiPS cells in vitro. (**A**) Four days of treatment with 0.1, 1 and 10 µg/mL ganciclovir (GCV). All sorted HSV-TK-expressing kiPS cells were eliminated while control cells (kiPS WT and kiPS GFP) remained intact despite mild non-specific toxicity in highest GCV dose. (**B**) Four days of treatment with 0.1, 1 and 10 µg/mL ganciclovir (GCV). Almost all unsorted HSV-TK-expressing kiPS cells were eliminated while control cells (kiPS WT and kiPS GFP) remained intact. All images: magnification 100×, and white bars represent 100 µm.

Our main goal was to introduce genetic modification allowing efficient elimination of transplanted cells upon their undesired behavior. Administration of prodrug non-toxic for normal cells would cause elimination of all transplanted cells and thus increase safety of iPS in clinical application. Genetically modified, HSV-TK expressing cells were highly sensitive to all applied doses of ganciclovir (Figure 5A,B). After only four days of 0.1 µg/mL ganciclovir treatment, virtually all HSV-TK expressing cells were eliminated in vitro proving high efficiency of applied suicide gene therapy. It has to be noticed that, apart from mild non-specific toxic effect of 10 µg/mL ganciclovir, no cytotoxic effect was observed in kiPS WT and kiPS GFP line suggesting high specificity of HSV-TK+GCV system (Figure 5A). Moreover, thanks to described bystander effect, unmodified cells within unsorted population of kiPS HSV-TK cells were also efficiently eliminated within four days. Figure 5B illustrates that all cells in culture are efficiently eliminated when only 40% of cells in population express HSV-TK (unsorted kiPS HSV-TK population). These data are consistent with our previous observations in rhabdomyosarcoma, where only 20% of HSV-TK expressing cells in population were enough to eradicate basically every cell in the culture by ganciclovir treatment [29].

**Figure 6:**
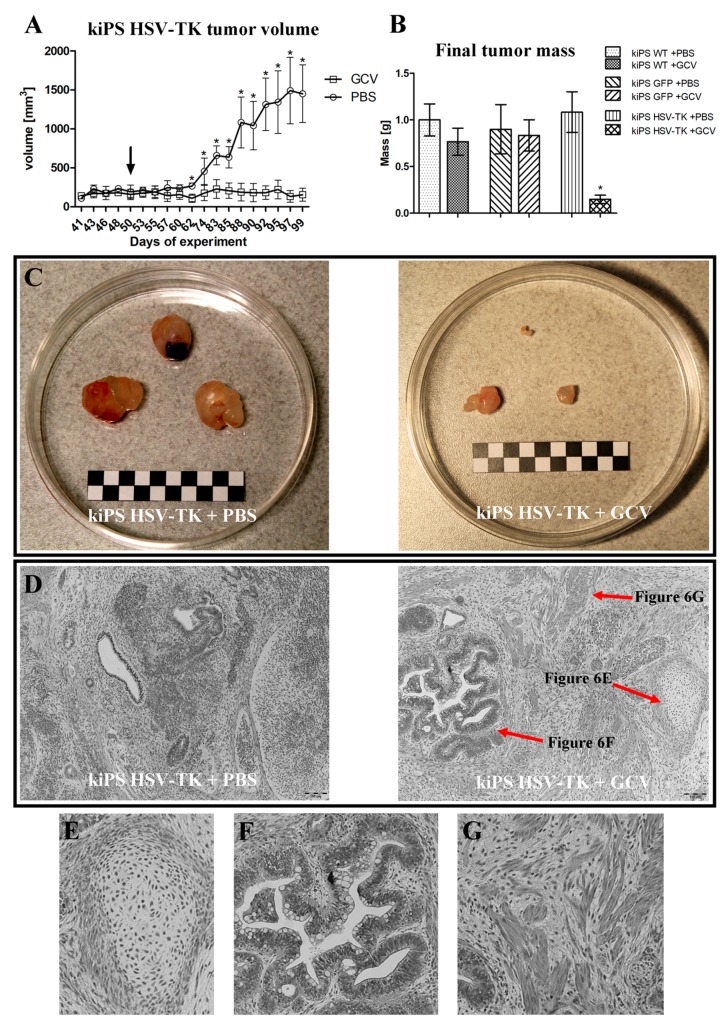
Ganciclovir efficiently eliminates tumors formed by HSV-TK expressing kiPS cells. After formation of tumors mice were daily injected with saline (PBS) or 50 mg/kg ganciclovir (GCV) intraperitoneally for 42 days. Growth of kiPS HSV-TK teratomas was abrogated after administration of ganciclovir (start of treatment is pointed by black arrow). (**A**) kiPS HSV-TK tumor volume during experiment; (**B**) final tumor mass; (**C**) dissected tumors; and (**D**) histopathologic images of kiPS HSV-TK-formed teratomas containing three germ layer derivatives: (**E**) cartilage; (**F**) secreting epithelium; and (**G**) muscle. Data in (**A**,**B**) are presented as mean ± standard error. * indicate statistically significant difference (*p* < 0.05) vs. appropriate control tested by Mann–Whitney *U*-test. Representative data for one of two replicates, in each *n* = 3. White and black squares in (**C**) represent 5 mm; and (**D**–**G**) magnification 100×, bars represent 100 µm.

The suicide gene therapy was also successful in in vivo experiments (Figure 6). Compared with control group (kiPS HSV-TK + PBS), size (Figure 6A) and weight (Figure 6B) of tumors formed by kiPS HSV-TK cells drastically decreased upon administration of GCV. Tumors formed by kiPS HSV-TK cells were almost completely eradicated upon systemic administration of GCV (Figure 6C). Moreover, no systemic toxicity was observed upon this treatment, showing the high specificity of the described approach. Growth of tumors formed by control cell line (kiPS WT and GFP) were not abrogated by administration of GCV (Figure 6B and Figure S1).

It is worth mentioning that genetic modification introducing HSV-TK expression did not alter any properties or quality of established kiPS. They retained characteristic morphology, growth rate, formed embryoid bodies and could form teratomas (Figure 6D). These observations suggest that their potential for differentiation is not compromised and their pluripotency is sustained. Performed genetic modification introducing exogenous expression of HSV-TK did not change pluripotency status or biology of generated kiPS cells.

Acquired results demonstrate high efficiency and specificity of described iPS suicide gene therapy, both in vitro and in vivo. Presented concept increases iPS cells safety for future clinical application. Introduction of a “safety switch” allows efficient elimination of transplanted cells upon previously unexpected necessity.

## 3. Discussion

iPS cells are a great promise for future regenerative therapies of many diseases. However, their pluripotency is also their greatest hazard, as their tumorigenic potential must be overcome before they will advance to clinic.

Here we show that introduction of genetic “trigger” allowing efficient elimination of all transplanted cells can increase safety of iPS cells in future clinical applications by safeguarding their main risk—tumorigenesis.

Our safeguarded kiPS cell lines were developed by reprogramming of plucked hair keratinocytes, an alternative and convenient source of somatic cells which can deliver cells for reprogramming in easy and non-invasive manner. iPS cells can be generated by reprograming virtually any somatic cell type. Most desirable are cells which can be acquired by non-invasive method. As harvesting skin fibroblast by biopsies can be painful and collecting blood samples is not always possible (patients’ needle phobia or viral infection), plucking hair for isolation of keratinocytes seems to be a legitimate option. Skin keratinocytes are known to be easier to reprogram than skin fibroblast because of their endogenous expression of *KLF4* and *c-MYC* [39].

Non-integrating, episomal viral vector based on Sendai virus [40,41] was used to reprogram acquired keratinocytes. This method provides safe and efficient reprogramming technique. Although our reprogramming efficiency (0.01%) was lower than described for reprogramming of neonatal fibroblast with Sendai virus [20] and keratinocytes with retroviral vectors [39], it appeared sufficient to deliver fully functional kiPS cell lines. Decreased efficiency of reprogramming might have been caused by suboptimal protocol of keratinocytes infection with Sendai virus (to our knowledge previously unpublished) and more differentiated/senescence state of keratinocytes acquired from hair plucked form adult individuals than neonatal cells [42].

Generated kiPS cells lines were efficiently genetically modified by lentiviral transduction to express transgene of interest—*HSV-TK*—and reporter gene—*GFP*. It has to be clearly noted that kiPS cells transduction did not interfere with their pluripotency as modified cells were able to efficiently form teratomas (Figure 6D), which proves their pluripotency [43]. Thus, genetic modification of kiPS cells is neutral for their biology involving growth rate, differentiation potential and pluripotency features. Unfortunately, use of lentiviral vectors for generation of genetically safeguarded iPS cells is not the optimal solution as it generates risk of insertional mutagenesis [44] which is not desired in clinical application of stem cells. Lentiviral vectors were used here as a method of choice for proof-of-concept studies. This impediment can be easily overcome by application of viral vector which specifically integrates into neutral location in the genome—e.g., so-called “safe harbor” on chromosome 19 in the case of adeno-associated viruses (AAV) [45]. Generation of suicide gene system involving AAV and CRISPR/Cas9 is goal of our ongoing study.

We showed that described suicide gene approach is very efficient in selective elimination of cells expressing HSV-TK. Virtually all genetically modified kiPS cells were eradicated within four days of treatment with low dose of GCV in vitro. In addition, in vivo tumors generated by kiPS HSV-TK cells were almost completely eliminated by low doses of GCV, without any systemic toxicity. 

In our bicistronic vector two transgenes were linked by IRES sequence, with *HSV-TK* as second cistron, which expression is known to be considerable lower than expression of the first gene [46]. kiPS HSV-TK cells were very efficiently eliminated by GCV in vitro, even compared to our previous experiments with rhabdmyosarcoma cells, despite weaker promoter applied here (UbC here vs. CMV in [29]). These observations suggest high vulnerability of iPS cells to toxic GCV metabolite generated by HSV-TK and feasibility of designed suicide gene therapy of iPS cells. Constitutive promoter for ubiquitin C applied in generated gene constructs ensures constant and long-term expression of transgenes in differentiated cells of all types. This is a trait of key meaning in designing life-long “emergency exit”. Another promising constitutive promoter is Elongation Factor-1-Alpha (EF-1-alpha) as it guarantees relatively strong and permanent expression of transgene [47]. 

In another approach, we plan to generate a vector in which expression of suicide gene is driven by promoter specific for embryonic/pluripotent cells (e.g., *NANOG* or *OCT4*). Such approach could allow eradication of any undifferentiated cells within differentiated cells population derived from iPS cells before their transplantation and would selectively sensitize putative de- or un- differentiated cells in transplants. These two tactics (suicide gene-based purification of cells before transplantation and specific safeguarding of transplanted cells) are other ideas to increase iPS cells’ safety. They can also be combined with approach described here by use of two separate *suicide genes* and promoters to generate “double emergency switch”. Deoxycytidine kinase and cytosine deaminase are examples of other suicide genes [48,49].

We presented selective elimination of human iPS cells by HSV-TK+GCV system. Safeguarding of primates iPS cells [49] and human ES cells [50] with suicide genes as well as numerous suicide gene therapies of cancer cells [27,28,29,30,31,32,48,51] including phase I clinical trial [52] were already described proving universal character of described approach.

iPS cells, thanks to their pluripotency, are great promise for future regenerative therapies of many diseases. However, their pluripotency is also their greatest risk, as their tumorigenic potential must be overcome before they will advance to clinic. Safety is key matter in regenerative therapy involving stem cells application.

## 4. Materials and Methods

### 4.1. Cell Cultures

Generated keratinocytes-derived induced pluripotent stem cells (kiPS) were routinely cultured on Gelatin (Sigma-Aldrich, Saint Louis, MO, USA) coated dishes with feeder layer of mitomycin C (Sigma-Aldrich)-inactivated mouse embryonic fibroblasts (MEFs) in serum-free iPS medium consisting of DMEM/F12 with 20% KSR, 2 mM GLUTAMAX, 100 µM Non-Essential Amino Acids, 100 U/mL/100 µg/mL Penicillin/Streptomicin, 10 ng/mL bFGF (all from ThermoFisher Scientific, Waltham, MA, USA) and 100 µM β-mercaptoethanol (Sigma-Aldrich) until confluent. Medium was changed every day. kiPS cells were subcultured with Accutase (Lonza, Basel, Switzerland) in proportion 1:4–1:10 and seeded onto fresh feeder layer in presence of 10 µM ROCK inhibitor Y-27632 (Sigma-Aldrich). For transduction and cell sorting experiments kiPS cells were cultured in feeder-free conditions on growth factor-reduced Matrigel (Corning, New York, NY, USA)-coated dishes in MEF-conditioned iPS medium. kiPS cells were cryopreserved in freezing medium consisting of 90% FBS (Eurx, Gdańsk, Poland), 10% DMSO (Sigma-Aldrich), 10 µM Y-27632 in density of 1.5 × 10^6^ cells/mL and after 1 h pre-incubation with 10 µM Y-27632.

MEFs were cultured in Dulbecco’s Modified Eagle’s Medium (DMEM) containing 4.5 g/L glucose (Lonza) supplemented with 10% *v*/*v* FBS, 2 mM L-glutamine and 100 U/mL/100 µg/mL Penicillin/Streptomycin antibiotics solution (all from ThermoFisher Scientific). Keratinocytes were cultured in serum free EpiLife medium (ThermoFisher Scientific) with 100 U/mL/100 µg/mL Penicillin/Streptomycin. All cells were cultured at 37 °C in a humidified atmosphere of 5% CO_2_.

### 4.2. Isolation of Keratinocytes from Plucked Hair

Keratinocytes were isolated from outer root sheath (ORS) of the plucked hair by direct outgrowth according to the protocol described before [53]. Experimental protocol was approved by Jagiellonian University Bioethical Committee in Kraków by decision number KBET/173/B/2012. Briefly, hairs plucked with ORSs form occipital part of the head were rinsed with antibiotic/antimycotic solution (ThermoFisher), cut and placed onto growth factor-reduced Matrigel (Corning) coated dishes in small amount of MEF-conditioned iPS medium. On the following days, small portions of medium was added until large outgrowths of keratinocytes surrounding the hair could be observed. At this stage medium was changed to EpiLife and cells were subcultured with TrypLE (ThermoFisher Scientific) and expanded on Matrigel-coated dishes until reprogramming.

### 4.3. Keratinocytes Reprogramming into iPS Cells

Isolated keratinocytes were infected with Sendai virus-based CytoTune—iPS Reprogramming kit (ThermoFisher Scientific) [20] according to fibroblast reprogramming manufacturer’s instructions as follows: 3 × 10^5^ keratinocytes were infected in confluency of 40–60% in Multiplicity of Infection, MOI of 5:5:5:3 *(KLF4:OCT4:SOX2:C-MYC*). Twenty-four hours post infection and every other day until Day 6 post infection, medium was replaced with fresh EpiLife medium. On Day 7 post infection, cells were seeded onto 100 mm culture dish with gelatin and mitomycin-C inactivated MEF feeder layer in iPS medium containing 1 mM sodium butyrate (Sigma-Aldrich). From this point, cells were cultured in 5% O_2_ and medium was changed daily. First emerging colonies could be observed on day 11. On day 15 sodium butyrate was withdrawn and feeder layer was renewed with fresh mitomycin-C inactivated MEFs. Between 18 and 35 days after infection, colonies with typical iPS morphology and expression of alkaline phosphatase (investigated by LiveStain, ThermoFisher Scientific) were manually transferred with a pipette into separate dishes with fresh feeder layers in iPS medium containing 10 µM Y-27632. Medium was changed daily until transferred colonies were large enough to be subcultured with Accutase. A total of 18 colonies were transferred.

### 4.4. Immunocytochemistry

Cells were rinsed with PBS with Ca^2+^ and Mg^2+^ (Lonza) and fixed in 4% paraformaldehyde (Sigma-Aldrich) in PBS with Ca^2+^ and Mg^2+^ (Lonza) for 20 min in room temperature. After paraformaldehyde removal cells were rinsed three times with PBS with Ca^2+^ and Mg^2+^ and permeabilized with 0.1% Triton X-100 (Sigma-Aldrich) in PBS with Ca^2+^ and Mg^2+^ (5 min in room temperature). Then the cells were rinsed and unspecific binding sites were blocked with 3% BSA in PBS with Ca^2+^ and Mg^2+^ (30 min in room temperature). The cells were incubated with appropriate antibodies diluted in 3% BSA in PBS with Ca^2+^ and Mg^2+^ overnight in 4 °C, rinsed three times with PBS with Ca^2+^ and Mg^2+^ and incubated with appropriate fluorescently labelled secondary antibodies and Hoechst (ThermoFisher Scientific) diluted in 3% BSA in PBS with Ca^2+^ and Mg^2+^ for 1 h in room temperature in the dark. Antibodies and dilutions were as follows: SSEA-3 1:100 (BD, Franklin Lakes, NJ, USA), SSEA-4, mouse 1:100 (BD, USA), TRA-1-60, rabbit 1:100 (BD), TRA-1-81, mouse 1:100 (BD, USA). Protein-iPS (piPS, System Biosciences, Palo Alto, CA, USA, [14]) were used as positive control.

### 4.5. RT-PCR

Gene expression on the level of mRNA was analyzed by RT-PCR. Briefly, total RNA was isolated with GeneMATRIX Universal RNA kit (Eurx). Reverse transcription (RT) was performed with M-MLV reverse transcription kit (Promega, Madison, WI, USA) according to manufacturer instructions. PCR reactions were performed with Taq PCR Master Mix (Eurx). One hundred nanograms (per reaction) of generated cDNA was used as a template. All used primers were designed to work optimally at annealing temperature of 55 °C. Sequences (5’ to 3’) of used primers: OCT4 for ATGGCGGGACACCTGGCTT, Oct4 rev GGGAGAGCCCAGAGTGGTGACG, NANOG for TGAACCTCAGCTACAAACAG, NANOG rev TGGTGGTAGGAAGAGTAAAG, TERT for TGTGCACCAACATCTACAAG, TERT rev GCGTTCTTGGCTTTCAGGAT, C-MYC for ATGCCCCTCAACGTTAGCT, C-MYC rev TTACGCACAAGAGTTCCG, GAPDH for CAAAGTTGTCATGGATGACC, GAPDH rev CCATGGAGAAGGCTGGGG, Primers sequence for Sendai virus genome (SeV) and transgenic c-Myc (vc-Myc) were given by manufacturer in Sendai virus kit manual. Protein-iPS (piPS, System Biosciences, [14]) were used as a negative control and freshly reprogrammed sendai virus-iPS (sv-iPS) as a positive control.

### 4.6. Teratoma Formation

All animal experiments were conducted according to ethical committee guidelines. All experimental protocols were approved by 2nd Local Institutional Animal Care and Use Committee (IACUC) in Kraków by decision number 162/2015 on 24 June 2015. Generated kiPS cells were subcutaneously injected into left dorsal flank of adult female NOD/SCID mice. Cells (3 × 10^6^) in 200 μL of PBS/growth factor-reduced Matrigel (Corning) 1:1 were injected per mice. Growth of tumors and mice health was monitored every other day.

### 4.7. Generation and Analysis of Genetically Modified Cells

Lentiviral particles were generated accordingly to ViraPower protocol (ThermoFisher Scientific). HEK293T cells (9.5 × 10^6^) were seeded in DMEM High Glucose, 10% FBS. Transfection with calcium orthophosphate was performed four hours after seeding. The transfection mixture was as follows: 26–39 µg of plasmid DNA, 65 µL of 2.5M CaCl_2_, 650 µL of 2× BBS (BES buffered saline, pH = 7.2) and 585 µL H_2_O. Transfection mix was incubated for 20 min in room temperature before transfer into fresh culture medium with 25 µM chloroquine (Sigma-Aldrich). Lentiviral particles containing medium was harvested 48 and 72 h after transfection. Harvested medium was centrifuged (3000 rpm, 10 min, 4 °C) and filtered through 0.45 µm syringe filter to eradicate remaining HEK293T cells and ultracentrifuged (25,000× *g*, 2 h, 4 °C) to concentrate the viral particles and transfer them to serum-free iPS cell medium. Transfection mixtures consisting of either GFP@pLenti6/Ubc or GFP-ires-HSV-TK@pLenti6/Ubc expression plasmid and pLP1, pLP2, pLP-VSVG packaging plasmids were prepared. Plasmids GFP@pLenti6/Ubc and GFP-ires-HSV-TK@pLenti6/Ubc were prepared by cloning GFP or GFP-ires-HSV-TK inserts from pLOX-gfp-ires-HSV-TK plasmid into Gateway pLenti system (ThermoFisher Scientific) according to manufacturer’s instructions. pLOX-gfp-ires-HSV-TK plasmid was a gift from Didier Trono (Addgene plasmid #12243). The same copy number (1.5 × 10^12^) of both expression plasmids were used. Maps of plasmid applied can be found in Figure S2.

To determine the titer of lentiviral stocks, HT1080 cells were infected. Four hours prior to transduction, 5 × 10^4^ cells were seeded on 24-well plate. Various volumes of condensed virus solutions (0.1–100 µL) were added to seeded HT1080 cells. Forty-eight hours after transduction the percentages of modified (GFP-expressing) cells were assessed by Attune flow cytometer (ThermoFisher Scientific) to calculate Transduction Units (TU) in 1 mL of each harvested medium.

To obtain modified cell lines, iPS cells cultured in feeder-free conditions were infected using generated lentiviral particles introducing GFP and HSV-TK gene in kiPS HSV-TK cell line or GFP only in kiPS GFP control cell line. Transduction was performed with MOI = 3 (Multiplicity of Infection) on 6-well plate. kiPS GFP and kiPS HSV-TK cell lines were selected with 5 µg/mL Blasticidin (ThermoFisher Scientific) for two weeks and purified using FACS Aria II cell sorter (BD). The percentage of green fluorescent cells in modified cell lines have been measured by Attune (ThermoFisher Scientific) flow cytometer.

### 4.8. Suicide Gene Therapy In Vitro 

First, 2.5 × 10^4^ of wild-type (WT) and genetically modified kiPS cells (GFP and HSV-TK) were seeded onto well of 24-well plate. Four hours after seeding, medium was changed for medium containing 0.1, 1 and 10 μg/mL ganciclovir or control fresh medium. All medias were changed daily. On each of following 5 days, cells in each group (WT, GFP and HSV-TK with or without GCV addition) were observed to analyze the influence of GCV.

### 4.9. Xenografts and Suicide Gene Therapy In Vivo

All animal experiments were conducted according to ethical committee guidelines. All experimental protocols were approved by 2nd Local Institutional Animal Care and Use Committee (IACUC) in Kraków by decision number 162/2015 on 24 June 2015. kiPS (WT, GFP and HSV-TK) cells were subcutaneously injected into left dorsal flank of adult female NOD/SCID mice. Cells (3 × 10^6^) in 200 μL of PBS/growth factor-reduced Matrigel (Corning) 1:1 were injected per mice. Tumors growth and mice health was monitored every other day. When tumors become measurable mice in each group (*n* = 6) were randomly divided into two regimes: receiving 1 mg of ganciclovir in 0.5 mL PBS (50 mg/kg of body weight) and receiving only 0.5 mL PBS (*n* = 3). Both groups were injected intraperitoneally, daily for 42 days. After this period, mice were sacrificed, tumors and internal organs (liver, kidney, spleen, lungs, brains and hearth) were isolated and fixed in 40% paraformaldehyde. Tumors were measured and weighed. Fixed tissues were imbedded in paraffin, cut, stained with hematoxylin/eosin and analyzed histopathologically. Volume (v) of tumors was calculated with equation v = 1/2 ab^2^, where a and b are measured tumor sizes.

### 4.10. Statistical Analysis

Statistical analysis of acquired data was performed with STATISTICA 10.0. Software (StatSoft, Tulsa, OK, USA). Mann–Whitney *U*-test or Kruskal–Wallis tests were applied to verify statistically relevant differences between experimental groups. Data are presented as mean ± standard error. Statistically significant differences (*p* < 0.05) were marked with *. 

## 5. Conclusions

We showed that iPS cells can be conveniently acquired by safe reprograming of plucked-hair keratinocytes. Generated in patient-specific manner iPS cells can be genetically secured with *suicide gene*, which introduces an “emergency exit” allowing easy, fast and efficient eradication of transplanted cells upon their undesired behavior. We believe that described approach increases safety of iPS cells and brings them one step closer to clinical application.

## Supplementary Materials

Supplementary materials can be found at www.mdpi.com/1422-0067/19/1/197/s1.

## Abbreviations

iPS: induced Pluripotent Stem cells
kiPS: eratinocytes-derived-iPS
piPS: protein-iPS
HSV-TK: Herpes Simplex Virus Thimidine Kinase
GCV: Ganciclovir
GFP: Green Fluorescence Protein
