# Supplementary material for: Introduction of Exogenous HSV-TK Suicide Gene Increases Safety of Keratinocyte-Derived Induced Pluripotent Stem Cells by Providing Genetic “Emergency Exit” Switch
Source: Int J Mol Sci. 2018 Jan 9;19(1):197. doi: 10.3390/ijms19010197 (PMC5796146; doi:10.3390/ijms19010197)
Supplement: Supplementary file 1 [file ijms-19-00197-s001.pdf]

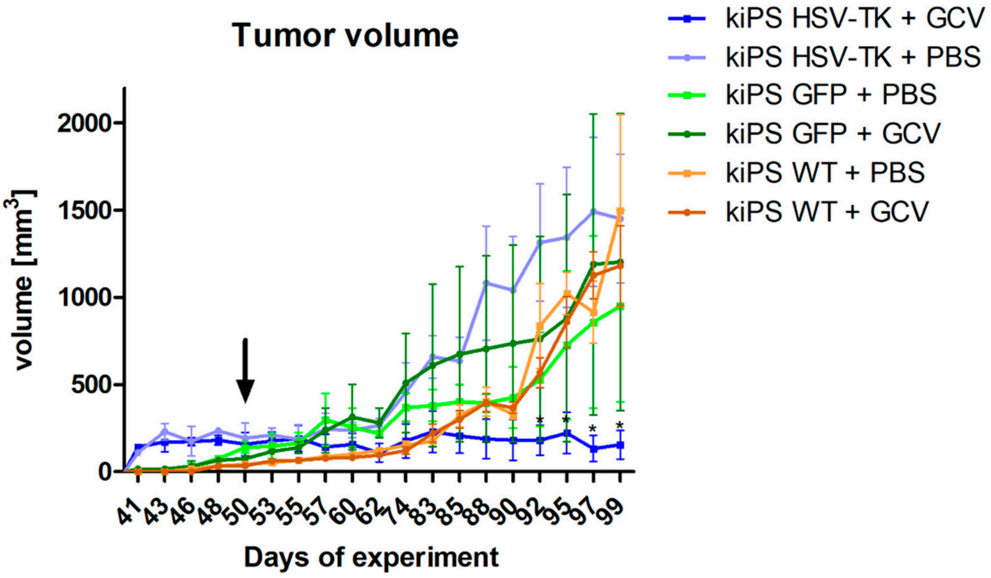

Figure S1. Ganciclovir selectively eliminates tumors formed by HSV-TK expressing kiPS cells. After formation of tumors, mice were daily injected with saline (PBS) or 50 mg/kg ganciclovir (GCV) intraperitoneally for 42 days. Only growth of kiPS HSV-TK teratomas was abrogated after administration of ganciclovir (start of treatment is pointed by arrow). Tumor volumes during experiment are presented as mean ± standard error. * indicate statistically significant difference ($p < 0.05$) tested by Kruskal–Wallis test.

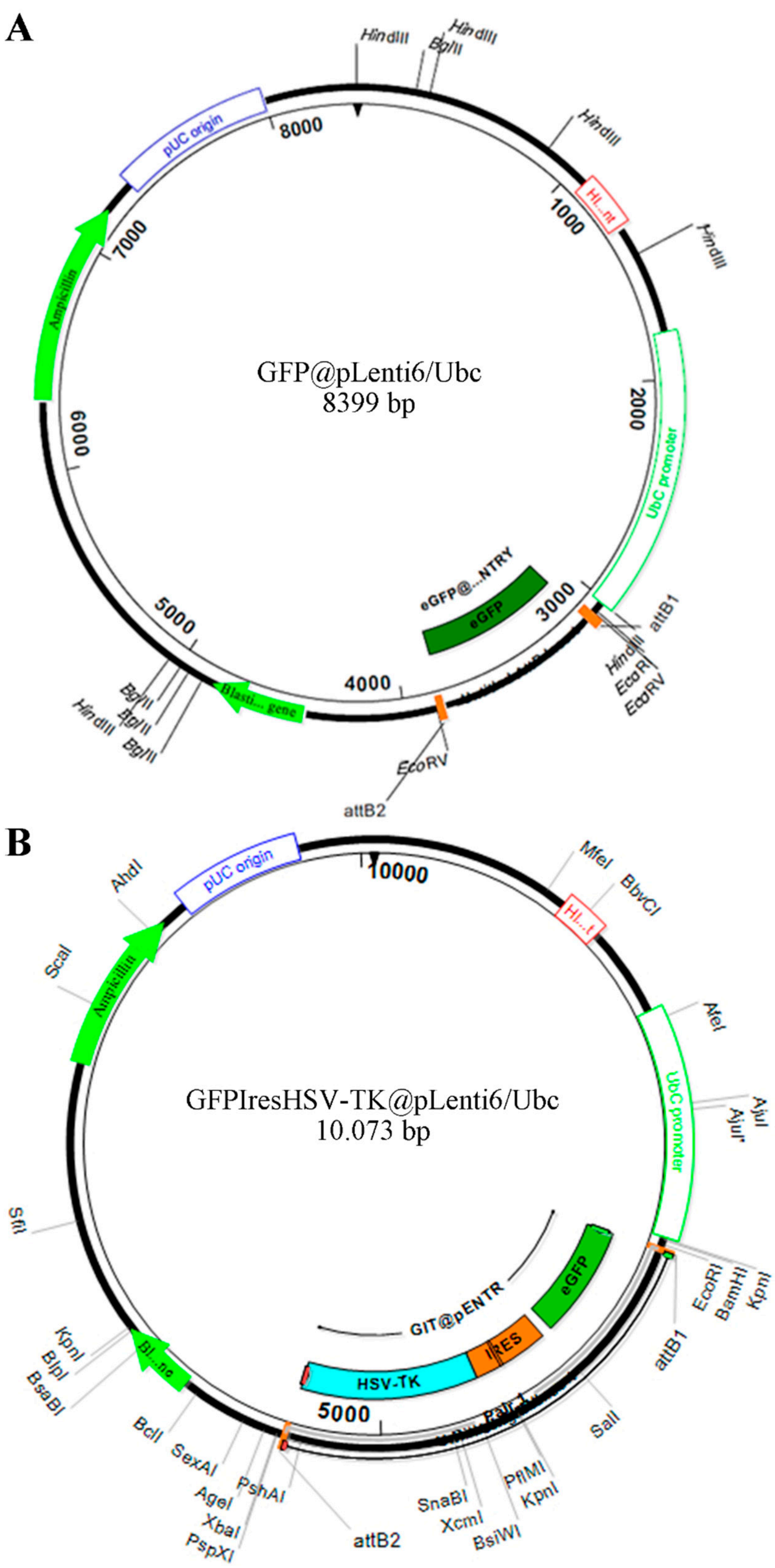

Figure S2. Maps of plasmids used to prepare lentiviral vetors: (**A**) map of plasmid GFP@pLenti6/Ubc; and (**B**) map of plasmid GFP-ires-HSV-TK@pLenti6/Ubc.
